# Removal of an Ovarian Tumor, in Which the Écraseur Was Used

**Published:** 1858-07

**Authors:** John L. Atlee

**Affiliations:** Lancaster, Pa.


### ART. II.—*Removal of an Ovarian Tumor, in which the Écraseur was used.* By JOHN L. ATLEE, M.D., of Lancaster, Pa.

On the 29th of January, 1858, I was called to see Mrs. E. E., of this city, in consultation with Dr. James Rodgers. She was then in her sixty-first year; commenced menstruation at seventeen; married at the age of twenty-three, and had never conceived. Her menses continued until the age of fifty-two, with occasional slight intervening leucorrhœa, and had always been more or less painful, and somewhat profuse. On one occasion, about two years after her marriage, she had uterine hemorrhage, which weakened her and kept her confined to bed for a few days. Soon after the cessation of the menses, she had an attack of erysipelatous inflammation of the face; since which time, until the winter of 1856–7, her health had been very good. Her husband died four years ago. Sometime in January or February, 1857, she became troubled with flatulent distension, as she supposed, of the lower portion of the bowels, and a frequent inclination to pass urine, although the quantity discharged was trifling. She had also occasional pain on each side, just above the groins, which resembled the pains of menstruation. On the 5th of August, finding her symptoms increase, she consulted Dr. Rodgers, who, upon examination, discovered a tumor occupying the whole lower portion of the abdomen, with evident fluctuation. A course of diuretic and hydragogue cathartic treatment, combined with mercurials, was instituted, and perseveringly carried out by him, alone, and subsequently in consultation with a highly respectable and experienced physician, with no other effect than to debilitate the patient. The size of the abdomen gradually increased; and on two occasions Dr. Rodgers was of the opinion, from the severity of the pain, that peritoneal inflammation existed in the left iliac region. The patient now complained so much of the discomfort arising from the distension that, on the 17th of December, her physicians removed eight pounds of a highly albuminous fluid by tapping. A tumor of considerable size remained, occupying the left hypogastric, iliac, and lumbar regions. The character of the disease being now understood, all active treatment was suspended, and the patient placed upon a more generous diet. The only medicine given was a mild saline aperient, to regulate the bowels. Four and a half weeks after the first tapping, a similar amount of fluid was drawn off by Dr. Rodgers. Two weeks after this, I was requested to see her. I found the abdomen as large as at the full period of pregnancy. There was evident fluctuation over the whole of the right side of the abdomen, extending to the left hypochondriac and upper portion of the lumbar regions. A very hard tumor occupied the lower portion of the left lumbar and iliac regions, pressure over which occasioned severe pain. Upon examination, per vaginam, I found the neck of the uterus occupying a central position in the pelvis; the left side presenting a tumor without fluctuation. On the right side a tumor could be felt, covering the brim, with evident fluctuation when the tumor was percussed from above. I diagnosed a multilocular tumor of the left ovary. Owing to the great oppression and pain arising from the pressure of the cyst, we tapped her again on the 3d of February, less than three weeks from the last operation, and eight and a half pounds of fluid were removed, containing so much albumen that it coagulated perfectly by heat.

This rapid filling of the cyst had caused great emaciation, and it was evident that unless resort was had to the removal of the cyst she could not long survive. Her pulse ranged from 90 to 100, and, occasionally, was above that; it was small and irritable, and quickened by mental emotion and exercise.

Three days after the last tapping a careful examination of the tumor was made, with reference to the practicability of an operation. The large remaining tumor, now that the abdomen was relaxed, could readily be moved to the opposite side; and inasmuch as the severe local pains, which existed when the abdomen was distended, were immediately relieved after tapping, I had reason to doubt the existence of much previous peritoneal inflammation, and consequent adhesion. After representing fairly and candidly all the dangers of the operation, in the presence of her attending physician and immediate friends, she was left to decide for herself,—my opinion having been given that it was practicable, and would be successful. At all events, should dangerous adhesions exist, the operation would be abandoned, and the wound closed; as had occurred to me in a previous case, with a favorable result. After various delays, and another tapping on the 3d of March, at which ten pounds of albuminous fluid were removed, she finally demanded the operation; being sensible, from the rapidity of the filling of the cyst, and her increasing debility and emaciation, that it could no longer be delayed. The day previous the bowels were freely evacuated, the patient restricted to cold water, and in the evening twenty drops of elixir of opium administered, to keep the bowels quiet. At midnight she took a second dose. Thirty drops were given on the following morning, the 23d of March, on which day, at noon, I proceeded to the operation, in the presence of Drs. Rodgers, Parker, Parry, Ehler, M. M. Nithens, and John L. Atlee, Jr., and Messrs. Weiger, Brensman, and Frick, medical students. The temperature of the room had been elevated to 80° F., and it was steadily maintained at that for several subsequent days. The bladder having been evacuated before placing her on the table, she was put under the influence of ether and chloroform, and an incision was made through the skin, cellular and adipose tissues, to the fascia, from one inch below the umbilicus to two inches above the pubis. The fascia and peritoneum were then successively divided upon the director to the extent of the external wound. The large cyst was now exposed, occupying the upper, lower, and right side of the abdomen, presenting, on its left aspect, a deep sulcus between it and a second cyst, which filled a large portion of the left lumbar and iliac regions. The hand was then introduced, and the adhesions caused by the previous tappings separated, as were also a few between the right abdominal wall and the cyst. It was then swept across the fundus of the cyst, and passed down between it and the omentum, so as to detach it in the few places where slight adhesions existed. Trochars were now introduced, and both cysts rapidly evacuated, the smaller one containing about four pounds of a straw-colored and less viscid fluid. Several smaller cysts, two or three times as large as the fist, were developed in the base of the tumor, rendering an enlargement of the wound, one inch at each extremity, necessary, before it could be drawn out. No other adhesions were found, except at the pedicle. The tumor, in its development, had deviated from the usual course. It had expanded the broad ligament, and encroached upon that portion of the peritoneum existing between it and the colon, so as to leave the pedicle but one inch long, four inches broad, and highly vascular, for the application of the ligature. In conversation with my brother, Dr. Washington L. Atlee, in February last, he had recommended the "écraseur" for severing the pedicle, and, as I had seen at least one fatal result from the application of the silk ligature to the pedicle too near to the colon, and as the use of the silver ligature of Dr. Sims, although less irritating than silk, would leave the proximal portion of the pedicle a sloughing, irritating mass within the abdomen, I determined to use the écraseur. The whole tumor being firmly drawn up and held steadily by my son, I surrounded the pedicle with the forefinger and thumb of the left hand, and grasped it very firmly. The chain of the instrument was now passed round it above the hand and very close to the tumor, and moderately tightened. By maintaining this position of the left hand very firmly—the screw of the instrument being held by an assistant—I was enabled to operate upon the lever with the right, and to prevent the colon from being drawn toward the chain. The lever was turned for half a minute, then stopped for half a minute, and so alternately until the pedicle was severed, occupying six and a half minutes. (The colon had entirely escaped, with a margin of peritoneum one inch wide.) The torn surface of the pedicle was then carefully sponged and examined; the abdomen was kept closed for several minutes, and no hemorrhage, nor even oozing of blood from it, could be detected. The fluid which had escaped from the cysts was then sponged out, and the external wound closed by four silver sutures (kindly sent to me by Dr. J. Marion Sims) and adhesive strips, supported by a compress and flannel bandage. During the whole operation the patient suffered but little pain; her pulse, which was 90 at the commencement, remained so when she was put to bed; the only variation was at one period, when, for a few moments, it became sensibly weaker, and a slight paleness overspread her countenance. There was no nausea or vomiting then, nor at any subsequent period. The whole weight of the mass—solid and fluid contents—was seventeen and a half pounds. Three hours after the operation the patient complained of a *screwing* pain low down in the left side, and occasionally a shooting pain in the same region. Pulse 86, and slightly intermittent; nose cold; skin of arms and hands warm, and a little moist. Has taken nothing but ice and iced water; at two o'clock she took thirty drops of the elixir of opium, having taken thirty when put to bed at half-past twelve o'clock.

5 o'clock, P.M. Patient complains of a strong inclination to pass water. Introduced catheter, and drew off sixteen ounces of straw-colored urine with acid reaction,—more, she says, than she had passed altogether during the previous week. Pulse 92, intermitting every eight or ten strokes; has less pain in left side; more in her back; slept nearly an hour; nose quite warm, as is the whole surface, with more fulness and quickness of the pulse; reaction evidently commencing.

7 o'clock, P.M. Patient now entirely free from pain; pulse 96; skin a little hotter than natural.

9½ o'clock, P.M. Patient quite free from pain; skin very moist; heat natural, except of palms, which are hot; catheter removed six ounces of urine; thirst considerable; at 8 o'clock took 25 drops elix. opii.; pulse 102; sleeps at intervals. Since the operation she has been more or less troubled with flatulence without pain.

Wednesday, March 24th, 8 o'clock, A.M. The report this morning is highly favorable; the patient smiled when I entered the room, and says she feels very well; she slumbered nearly all night, waking at intervals and asking for drink; she has no pain, and the only complaint she makes is that the wind is *rumbling* in her bowels; pulse 84, soft, and has lost its quickness; skin, including palms and soles, perfectly natural; perspired all night. It appears as if the reaction, which commenced at 5 o'clock yesterday, has entirely subsided, and left her with a better pulse than I have known her to have since I commenced my attendance upon her. Removed by the catheter eight ounces of urine; mind calm, clear, and hopeful; she completed her sixty-first year on the 8th inst.

12½ o'clock, P.M. Patient in all respects the same, except pulse, which is 90; to continue cold water and ice, *pro re nata.*

7½ o'clock, P.M. Patient still lively and comfortable; pressure on the abdomen in the left iliac region gives some pain, or feeling of soreness; has passed wind from the bowels this afternoon; bladder troublesome; removed, by the catheter, ten ounces of urine, of normal color and acid reaction; pulse 79. The intermission in the pulse, remarked yesterday, passed off during the night, and the action of the heart has been perfectly regular all day; tongue clear, skin moist, and of natural temperature; to have 25 drops elix. opii.; allowed thin arrow-root gruel during the night.

From this time until the 27th of March, 5th day, no unfavorable symptoms were manifested; the patient, on the contrary, steadily improving; examined the wound, and found it united throughout the whole extent, the lint covering it as clear as when first applied. There had been no serious discharge from the abdomen, as in all my other cases where the ligature was removed; no trace of inflammation around the silver sutures, which were removed on the 7th day; on this day she sat up for the first time out of bed.

From this time my patient sat up daily; on the 9th day the bowels were moved, for the first time, by a dose of castor oil; the discharges consistent and natural; she had been gradually allowed a more generous diet, and was increasing in strength.

On the following Monday, the 14th day, she rode with me half a mile, to my office, to see the tumor, and since then she has remained perfectly well.

This case is the eighth on which I have operated for the removal of enlarged ovaria, in two of which both the ovaria were diseased and removed, one only proving fatal, the patient dying on the 12th day. In all the previous cases I could perceive the disturbing effect of the presence of the silk ligature around the pedicle, as well as of those used to restrain hemorrhage, and until they came away they were more or less a source of anxiety both to the patients and to myself. With the écraseur to sever the pedicle, and the use of the silver suture and silver ligature, where ligatures are necessary, I am satisfied that a very large share of the dangers attending the operation will be obviated. Mrs. E. is the oldest patient upon whom, I believe, the operation has been performed,—Dr. Clay, of Manchester, England, reports his two oldest at 57 and 58; she was excessively reduced by the frequent tapping and refilling of the cyst, as well as by the previous very active medical treatment to remove the dropsy, and yet she recovered more rapidly and with much less constitutional disturbance than any other patient upon whom I have ever performed a capital operation. This I attribute very much to the use of the écraseur and the absence of any ligature involving the peritoneum.

